# Carbazolyl Electron Donor and Pyridinyl Electron Acceptor Containing Derivatives as Potential Host Materials for Green Organic Light-Emitting Diodes

**DOI:** 10.3390/molecules30091911

**Published:** 2025-04-25

**Authors:** Raminta Beresneviciute, Anil Kumar, Dovydas Blazevicius, Sushanta Lenka, Song-Ting Hsieh, Ming-Feng Tsai, Gintare Krucaite, Daiva Tavgeniene, Jwo-Huei Jou, Saulius Grigalevicius

**Affiliations:** 1 Department of Polymer Chemistry and Technology, Kaunas University of Technology, Radvilenu Plentas 19, LT50254 Kaunas, Lithuaniadovydas.blazevicius@ktu.lt (D.B.); gintare.krucaite@ktu.lt (G.K.); daiva.tavgeniene@ktu.lt (D.T.); 2 Department of Materials Science and Engineering, National Tsing Hua University, No. 101, Section 2, Guangfu Rd., Hsinchu 30013, Taiwan; anilgpchkee@gmail.com (A.K.); sushantalenka1@gmail.com (S.L.); ktpssr@gmail.com (M.-F.T.); 3 Department of Chemistry, National Tsing Hua University, No. 101, Section 2, Guangfu Rd., Hsinchu 30013, Taiwan; songtinghsieh@gapp.nthu.edu.tw

**Keywords:** bipolar derivative, thermal stability, green emission, glass transition temperature, organic light-emitting diode, efficiency

## Abstract

Here, we present two series of new electroactive compounds containing electron donors (carbazolyl) and electron acceptor (pyridinyl) fragments as potential host materials. The objective compounds 9-(2-ethylhexyl)-3,6-di [3-(methoxypyridin-3-yl)carbazol-9-yl]carbazoles **RB71** and **RB74** were synthesized by an Ullmann coupling reaction between the intermediate derivatives: 9-(2-ethylhexyl)-3,6-diiodocarbazole and corresponding 3-(methoxypyridin-3-yl)-9*H*-carbazole. Other target derivatives, 9-alkyl-3-[*N*-(9-alkylcarbazol-3-yl)-*N*-(4-methylpyridin-2-yl)amino]carbazoles **RB70** and **RB75**, were also prepared, according to the Ullmann reaction method, from 2-amino-4-methylpyridine and the corresponding 3-iodo-9-alkylcarbazole. Thermogravimetric analysis confirmed that the new derivatives are highly thermally stable compounds, with 5% weight loss in the temperature range of 349 °C to 488 °C. According to differential scanning calorimetry results, some amorphous materials exhibit very high glass transition temperatures exceeding 150 °C in some cases, which is a significant advantage for compounds with potential applications in organic light-emitting devices. The electroluminescent properties of devices utilizing the new hosts **RB71** or **RB70** with 5.0, 10.0, 15.0, and 20.0 wt.% concentrations of the dopant tris(2-phenylpyridine)iridium(III), Ir(ppy)_3_, were demonstrated. All the PhOLEDs emitted light at approximately 515 nm with CIE coordinates of (0.30, 0.61) due to Ir(ppy)_3_ emissions. The most efficient device with **RB71** host demonstrated a maximum power efficacy of 8.0 lm/W, maximum current efficiency of 12.7 cd/A, and maximal external quantum efficiency of 5.4% with a relatively low turn-on voltage of 4.3 eV, as well as luminance exceeding 4000 cd/m^2^. Additionally, 15 wt.% Ir(ppy)_3_ emitter-based PhOLED with **RB70** host outperformed the other devices by displaying a maximum power efficacy of 9.6 lm/W, maximum current efficiency of 16.0 cd/A, and maximal external quantum efficiency of 6.7% with a relatively low turn-on voltage of 3.7 eV, as well as luminance reaching 11,200 cd/m^2^. Some devices seem to exhibit higher efficiencies than those previously reported for OLEDs that utilize a 4,4′-bis(9-carbazolyl)-2,2′-biphenyl (CBP) host.

## 1. Introduction

Organic light-emitting diodes (OLEDs) are a multilayer technology wherein thin layers of charge-transporting and specific light-emitting organic derivatives are utilized [1]. The formation of various organic electroactive materials between two electrodes is a key procedure in the fabrication of OLEDs [2,3]. The development and synthesis of the novel materials for the charge transport layer, blocking layer, emitting layer, and more, result in relevant improvements in the performance of the devices [4]. The large fabrication areas [5], high performance [6], flexibility [7], high image quality [8], and low power [9] of the devices were successfully inducted for displays [10,11] and lightning technologies [12,13]. OLEDs are mercury-free and more efficient than fluorescent tubes, potentially becoming the next generation of general lighting [14,15].

Traditional fluorescent OLEDs use only singlet excitons for the light generation, which limits their maximum internal quantum efficiency to 25%. In contrast, the new generation of phosphorescent organic light-emitting diodes (PhOLEDs) shows more promise, because they can use heavy atom effects to generate both singlet and triplet excitons for emission and reach an internal quantum efficiency of 100% [16,17,18]. Among all the emitting phosphorescent complexes, iridium(III) complexes are regarded as the most feasible emitters for the PhOLEDs due to their high thermal stability, short excited-state lifetime, and strong spin–orbital coupling effect of the heavy metal atoms, which helps to achieve high quantum efficiency [19,20,21]. However, to remove harmful effects in the thin layers of the phosphorescent emitters, such as triplet–triplet annihilation and/or aggregation quenching (which usually are the main issues that decrease the device efficiency), a host–guest strategy of homogeneously dispersing the triplet guests into a suitable host material is frequently used [22,23,24]. Therefore, the design and synthesis of suitable hosts are essential for high-efficiency PhOLEDs. A suitable host derivative should have a higher triplet energy level than that of the phosphorescent emitter to ensure efficient energy transfer, high thermal stability, and good thin layer-forming properties, as well as suitable molecular orbitals for the facilitation of carrier transport in the device and a reduction in operating voltage [25,26,27]. As compared with unipolar hosts, bipolar hosts have an electron-rich donor and an electron-deficient acceptor in their structure, which increases OLED stability and enhances its performance by balancing electron and hole transport in the emitting layer [25,26,27]. Earlier, several series of donor–acceptor type derivatives containing hole-transporting fragments of triphenylamine [28,29,30], phenoxazine [27,31], or carbazole [25,26] were synthesized and tested in devices. Amongst them, carbazole is well known as a suitable donor fragment with high chemical stability, a special rigid structure, and high triplet energy [32,33,34]. In recent years, several series of pyridine rings as donor fragments containing electron-transporting compounds have been described as components for hole/exciton blocking and electron-transporting layers, enhancing the external quantum efficiency of the devices [35,36,37,38]. Also, polypyridine and polypyridine vinylidene were investigated by Yamamoto and his coworkers long ago as polymeric materials having negative charge carrier characteristics [39]. These studies proved the applications of the pyridine-based derivatives as components of OLEDs. Hence, it is interesting to consider pyridine when designing the bipolar host materials. Combining the pyridine unit with continuous conjugation and using suitable electron-donating fragments to create prospective bipolar derivatives could be an efficient strategy for designing efficient host materials. Taking the mentioned advantages of carbazole and pyridine in the host structure, the novel potential host materials, namely, 9-(2-ethylhexyl)-3,6-di [3-(methoxypyridin-3-yl)carbazol-9-yl]carbazoles and 9-alkyl-3-[*N*-(9-alkylcarbazol-3-yl)-*N*-(4-methylpyridin-2-yl)amino]carbazoles were designed and synthesized. We introduced a pyridine moiety as an acceptor to facilitate electron transmission, and carbazole fragments were introduced as the donor groups, connecting with the unconjugated alkyl chains, which can improve film-forming properties of the derivatives. Experimental results showed that the materials exhibited good physical properties with high thermal stabilities and suitable triplet energies for green phosphorescent emitters. Therefore, devices using tris(2-phenylpyridine)iridium(III) (Ir(ppy)_3_) guest were fabricated. Some of the green PhOLEDs based on the above-mentioned new host materials achieved suitable electroluminescence performance, with a maximum power efficacy of 9.6 lm/W, maximum current efficiency of 16.0 cd/A, and maximal external quantum efficiency of 8.1%, as well as a luminance reaching 11,200 cd/m^2^.

## 2. Results and Discussion

**Scheme 1:**
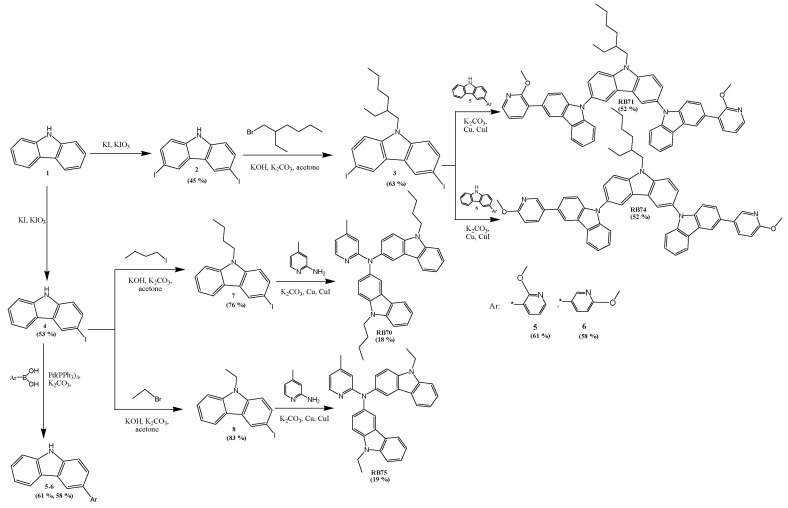
Synthetic pathway of materials **RB70**, **RB71**, **RB74**, and **RB75**.

The objective derivatives **RB70**, **RB71**, **RB74**, and **RB75** were prepared by a synthetic route, as shown in Scheme 1. Firstly, 3,6-diiodo-9*H*-carbazole (**2**) and 3-iodo-9*H*-carbazole (**4**) were prepared in the iodination reaction of 9*H*-carbazole (**1**) according to Tucker’s procedure. Then, intermediate carbazole derivatives, i.e., 9-butyl-3-iodocarbazole (**7**) and 9-ethyl-3-iodocarbazole (**8**), were synthesized as key materials from commercially available 1-bromoethane or 1-iodobutane, respectively, as well as 3-iodo-9*H*-carbazole (**4**) during alkylation reaction under basic conditions. Another alkylated compound, 9-(2-ethylhexyl)-3,6-diiodocarbazole (**3**), was produced in the reaction of 3,6-diiodo-9*H*-carbazole (**2**) with 2-ethylhexyl bromide as the alkylating agent. Intermediate carbazole derivatives 3-(2-methoxypyridin-3-yl)-9*H*-carbazole (**5**) and 3-(6-methoxypyridin-3-yl)-9*H*-carbazole (**6**) were prepared through the Suzuki reaction as it is described in the literature.

The objective compounds 9-(2-ethylhexyl)-3,6-di [3-(methoxypyridin-3-yl)carbazol-9-yl]carbazoles **RB71** and **RB74** were finally synthesized by the Ullmann coupling reaction between the 9-(2-ethylhexyl)-3,6-diiodocarbazole (**3**) and corresponding 3-(methoxypyridin-3-yl)-9*H*-carbazole (**5** or **6**). Other target derivatives, 9-alkyl-3-[*N*-(9-alkylcarbazol-3-yl)-*N*-(4-methylpyridin-2-yl)amino]carbazoles **RB70** and **RB75**, were also prepared according to the Ullmann reaction method from 2-amino-4-methylpyridine and corresponding 3-iodo-9-alkylcarbazole (**7** or **8**). Mass spectrometry (MS) and nuclear magnetic resonance spectroscopy (NMR) confirmed the newly prepared derivatives’ structures. The data showed good agreement with the proposed structures.

**Figure 1:**
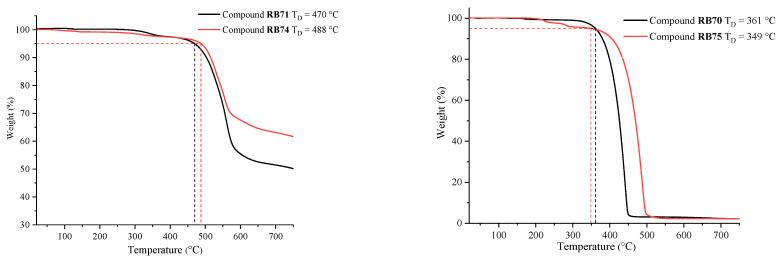
The curves of the TGA measurements of the materials **RB71**, **RB74** (**left**), and **RB70**, **RB75** (**right**).

The thermal characteristics of the prepared objective derivatives **RB71**, **RB74**, **RB70**, and **RB75** were established in a nitrogen atmosphere using differential scanning calorimetry (DSC) and thermogravimetric analysis (TGA). The curves of the TGA of these materials are demonstrated in Figure 1. The obtained results revealed that all these compounds have very high thermal stability. The temperatures of 5% weight loss (T_d_) for the prepared derivatives were, correspondingly, 470 °C for **RB71**, 488 °C for **RB74**, 361 °C for **RB70**, and 349 °C for **RB75**, as confirmed by the analysis using a heating rate of 10 °C/min. It could be seen that the materials **RB71** and **RB74** exhibited obviously higher values of T_d_ than those of derivatives **RB70** and **RB75**. This could be explained by the higher molecular weight of the branched molecules having three carbazole rings. The TGA curves of **RB70** and **RB75** demonstrate that these materials incur thermal evaporation during the experiment due to their lower molecular weight. Furthermore, it is essential to note that all the materials exhibited very high performance for their application in the thin layers of optoelectronic devices [40,41,42].

**Figure 2:**
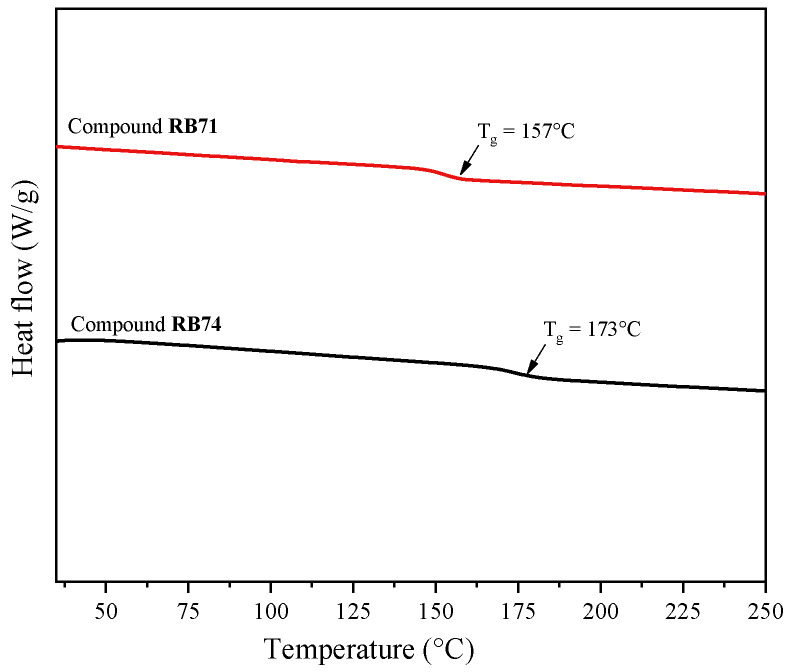
Curves of second heating in DSC measurements for compounds **RB71** and **RB74**.

The derivatives with three carbazolyl fragments, **RB71** and **RB74,** were obtained as amorphous materials after synthesis and purification by column chromatography, as confirmed by DSC measurement. The thermograms registered for the compounds **RB71** and **RB74** during their second heating cycle are presented in Figure 2. It is evident that these materials have very high glass transition temperatures (T_g_), which reach 157 °C for the derivative **RB71** and 173 °C for the material **RB74**. The high values of T_g_ are a significant advantage for the fully amorphous derivatives, with potential application in forming thin homogeneous electroactive layers in OLEDs, as they contribute to the enhanced long-term stability of the devices [43,44,45].

**Figure 3:**
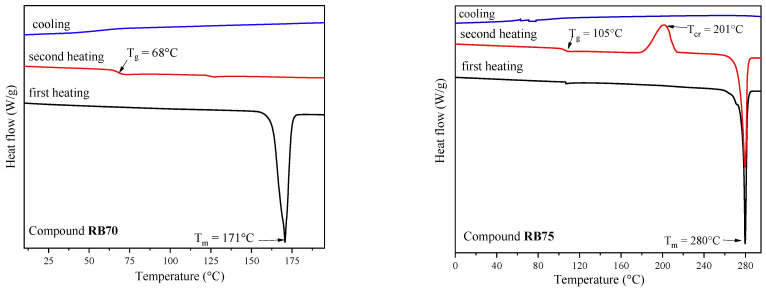
The curves of the DSC measurements of the compounds **RB70** (**left**) and **RB75** (**right**).

The lower molecular weight derivatives **RB70** and **RB75** with two carbazole rings showed different thermal transitions than those of the aforementioned fully amorphous materials. First, both compounds were obtained as crystalline materials after synthesis and purification by column chromatography; however, they could be transformed into amorphous materials by cooling the melted samples, as confirmed by the DSC experiments. The thermograms of the derivatives **RB70** and **RB75** are shown in Figure 3. When the crystalline material **RB70** was heated, an endothermic peak was observed at 171 °C due to melting. Then, the melted sample was cooled down and converted into an amorphous material. During the second heating of the sample, only the glass transition temperature was registered at 68 °C, and upon further heating, the sample showed no peaks associated with crystallization or melting. The derivative **RB75** demonstrated a stronger tendency for crystallization. Firstly, an endothermic peak due to melting was observed at 280 °C when the crystalline sample was heated for the first time. Then, the melted sample was cooled down and converted into an amorphous material. During the second heating of the sample, the glass transition temperature was observed at 105 °C, followed by crystallization at 201 °C to form the same crystals, which were obtained by crystallization from the solution and melted at 280 °C. It could be stated that the thin amorphous films of the materials **RB70** and **RB75** will be less stable than those of the branched derivatives **RB71** and **RB74**; however, such materials demonstrating the formation of an amorphous phase in DSC measurements were also successfully applied as electroactive layers in OLED devices [46,47,48].

The low-temperature photoluminescence was registered for the new compounds to determine their triplet energies. It was established that 9-(2-ethylhexyl)-3,6-di [3-(methoxypyridin-3-yl)carbazol-9-yl]carbazoles **RB71** and **RB74** possess high triplet energy levels of about 2.79 eV. 9-alkyl-3-[*N*-(9-alkylcarbazol-3-yl)-*N*-(4-methylpyridin-2-yl)amino]carbazoles **RB70** and **RB75** have triplet energy levels of 2.77 and 2.84 eV, respectively. The fluorescence spectra of **RB71** and **RB70** are depicted in Figure S13 and Figure S14. The characteristics confirm that the synthesized compounds could be suitable host materials for green phosphorescent emitters.

**Figure 4:**
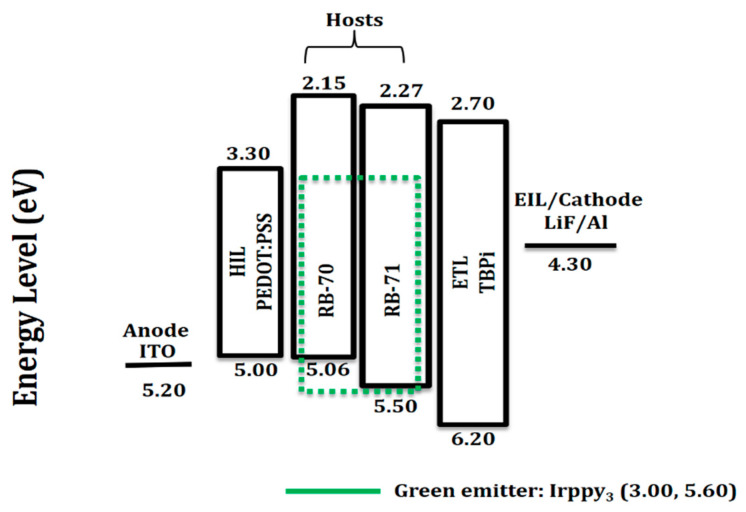
The energy level diagram in eV of the solution-processed green phosphorescent OLEDs containing hosts **RB71** or **RB70** with the phosphorescent emitter Ir(ppy)_3_.

Preliminary tests of the RB materials in the formation and characterization of phosphorescent OLEDs using the emitter tris(2-phenylpyridine)iridium(III), Ir(ppy)_3_, have demonstrated that the host materials **RB71** and **RB70** are the most promising components of the multilayer devices. Therefore, subsequent detailed investigations were conducted by using only the mentioned materials. The fabricated devices had the following structures: ITO (125 nm)/PEDOT: PSS (35 nm)/**RB71** or **RB70** host: (x wt.%) of Ir(ppy)_3_ emitter (x = 5.0, 10, 15 and 20%) (20 nm)/TPBi (40 nm)/LiF (1 nm)/Al (200 nm). The detailed device fabrication process is described in the Experimental Methods. Figure 4 presents the energy level diagram of the green light-emitting OLEDs using the hosts **RB71** or **RB70** doped with the green commercial phosphorescent emitter Ir(ppy)_3_. The HOMO-LUMO levels of the materials used in the devices were established by a procedure, which we have described earlier [49]. Cyclic voltammetry (CV) curves are depicted in Figures S15 and S16. It can be observed that both the host materials could be suitable for the mentioned dopant. However, the HOMO-LUMO levels of host **RB71** align more precisely with the corresponding characteristics of the green emitter.

**Figure 5:**
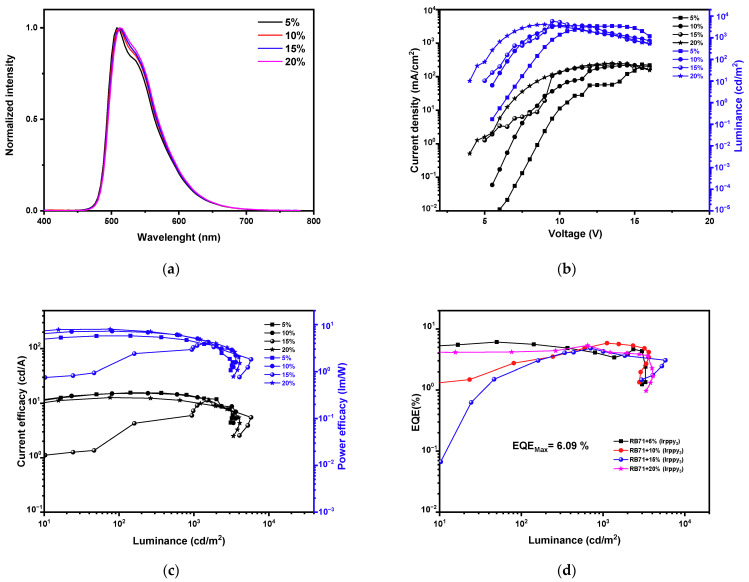
Characteristics of OLEDs using host material **RB71** with different concentrations of emitter Ir(ppy)_3_. (**a**) Electroluminescence spectra, (**b**) current density–voltage–luminance, (**c**) current efficacy–luminance–power efficacy, (**d**) external quantum efficiency–luminance.

The electroluminescent (EL) properties of the devices using the new hosts **RB71** or **RB70** with 5.0, 10.0, 15.0, and 20.0 wt.% concentrations of dopant Ir(ppy)_3_ were demonstrated. Figure 5 illustrates the electroluminescence spectra, current density–voltage–luminance, current efficacy–luminance–power efficacy, and external quantum efficiency–luminance characteristics of the OLEDs using the host material **RB71**. Some characteristics of these devices are also summarized in Table 1. The registered EL spectra (Figure 5a) exhibit very similar emission peaks for different doping concentrations in the **RB71** host-based devices. All the PhOLEDs emit light at about 515 nm due to the emissions of Ir(ppy)_3_. They do not have any color shift while changing the concentration of the green-emitting dopant. The fabricated devices emitted green light with CIE coordinates of (0.30, 0.61) and broad spectra from 460 to 700 nm. The presence of the single emission peak indicates the complete host–guest energy transfer and confirms that this host is well suited for the Ir(ppy)_3_ dopant.

The **RB71**-based PhOLEDs demonstrated turn-on voltages ranging from 4.3 to 7.2 V, maximal current efficiencies of 11.4–15.6 cd/A, maximal power efficiencies of 4.2–8.0 lm/W, maximal external quantum efficiencies of 4.8–6.1%, and maximal luminance in the range 3307–5795 cd/m^2^. At higher brightness, such as 1000 cd/m^2^, used for illumination applications, these OLEDs also exhibited promising performance with maximal current efficiencies of 10.5–13.50 cd/A, maximal power efficiencies of 4.2–5.3 lm/W, and maximal external quantum efficiencies of 2.3–4.8%. It could be stated that the 20 wt.% Ir(ppy)_3_ emitter-based device slightly outperformed the other PhOLEDs by displaying a maximum power efficacy of 8.0 lm/W, maximum current efficiency of 12.7 cd/A, and maximal external quantum efficiency of 5.4% with a relatively low turn-on voltage of 4.3 eV, as well as a luminance exceeding 4000 cd/m^2^.

**Figure 6:**
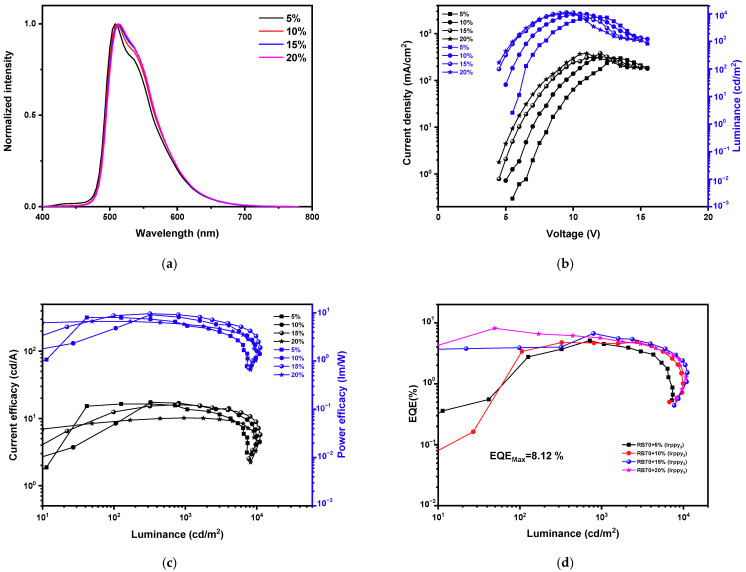
Characteristics of OLEDs using host material **RB70** with different concentrations of emitter Ir(ppy)_3_. (**a**) Electroluminescence spectra, (**b**) current density–voltage–luminance, (**c**) current efficacy–luminance–power efficacy, (**d**) external quantum efficiency–luminance.

The host derivative **RB70** was also employed to form the analogous green PhOLEDs in concentration-dependent experiments, where the amount of Ir(ppy)_3_ dopant ranged from 5 to 20 wt.%. Figure 6 presents the electroluminescence spectra of the devices as well as the current density–voltage–luminance, the current efficacy–luminance–power efficacy, and the external quantum efficiency–luminance characteristics of the PhOLEDs utilizing the host derivative **RB70** in the emitting layer. Some parameters of these devices are also listed in Table 2. All the electroluminescence spectra of the PhOLEDs (Figure 6a) exhibit quite similar emission peaks across different doping concentrations in the **RB70** host compound-based devices, which emit green light from the dopant Ir(ppy)_3_ at approximately 515 nm. All the spectra are quite similar, with CIE coordinates of approximately (0.30, 0.61), and there is no color shift when changing the concentration of the green guest material. These single peaks confirm the suitable energy transfer from the host **RB70** to the Ir(ppy)_3_ guest and show that this host material is well suited for the green dopant.

The devices formed with host **RB70** showed relatively low turn-on voltages ranging from 3.5 to 5.4 V, maximum external quantum efficiencies of 4.8 to 8.1%, notably high maximum current efficiencies of 10.4–17.3 cd/A, and maximum brightness values exceeding 7400–11,200 cd/m^2^. The high current efficiencies and relatively low voltages give power efficiencies of 6.6–9.6 lm/W. At a brightness of 1000 cd/m^2^, which is required for illumination applications, these devices also had a promising performance with maximal current efficiencies of 10.2–16.5 cd/A, maximal power efficiencies of 5.6–8.9 lm/W, and maximal external quantum efficiencies of 3.0–4.8%. It could be declared that the 15 wt.% Ir(ppy)_3_ emitter-based PhOLED outperformed the other devices by displaying a maximum power efficacy of 9.6 lm/W, maximum current efficiency of 16.0 cd/A, and maximal external quantum efficiency of 6.7%, with relatively low turn-on voltage of 3.7 eV as well as luminance reaching 11,200 cd/m^2^. Additionally, the efficiencies of green phosphorescent devices seem to be higher than those of previously reported OLEDs that utilize 4,4′-bis(9-carbazolyl)-2,2′-biphenyl (CBP) host and 1,3,5-tris(1-phenyl-1H-benzimidazol-2-yl)benzene (TPBi) as the electron transporting material [50]. It is important to note that the performance of the PhOLEDs could be further improved by thoroughly optimizing the layered structures, formation conditions, and charge-transporting materials.

## 3. Experimental Methods

### 3.1. Instrumentation

Thermogravimetric analysis (TGA) was performed on a TGAQ50 apparatus (Verder Scientific Haan, Haan, Germany). The TGA and DSC curves were recorded in a nitrogen atmosphere at a 10 °C/min heating rate. Differential scanning calorimetry (DSC) measurements were carried out using a Bruker Reflex II thermos-system (Bruker, Berlin, Germany). Low-temperature photoluminescence (LTPL) spectra of the compounds were recorded in tetrahydrofuran solutions on a Hitachi F-7000 spectrophotometer (Edinburgh Instruments Ltd., Livingston, UK) with a delay time of 6.25 ms at a temperature of 77 K. The spectra were used to register triplet energy values of the derivatives at the onsets of the LTPL spectra.

The OLED devices were formed on pre-patterned ITO glass substrates, which were first cleaned with a soap solution for 10 min and then rinsed for 5 min with distilled water. Then, the substrates were ultrasonically cleaned for 30 min in acetone at 50 °C, and afterward, for a further 30 min, isopropyl alcohol was used at 60 °C. After the cleaning, the substrates were treated for 15–20 min with UV to remove the solvents, and then they were transferred to a nitrogen-filled glove box. Deposition of a multilayer OLED structure was carried out in the glove box under an inert atmosphere. Hole-injecting layer of PEDOT: PSS was spin-coated at 4000× *g* rpm for 20 s, and then the substrates were heated at 130 °C for 10 min and then cooled. An emissive layer was then spin-coated from the solution of RB host with the dopant Ir(ppy)_3_ on the cooled substrates at 2500× *g* rpm for 20 s. The substrates were then transferred to a thermal evaporation chamber for deposition of TPBi electron transporting and LiF injecting layer, as well as an Al cathode at a high vacuum of 10^6^ torr. The device area was 0.09 cm^2^.

The OLED devices were characterized in a completely dark room under ambient conditions. The current density–voltage–luminance characteristics were recorded using a CS-100A luminescence spectrophotometer (Becker & Hickl, Berlin, Germany), while power efficacy–luminance–current characteristics were recorded using a PR-655 spectrophotometer (NLIR, Farum, Denmark). The Keithley 2400 voltmeter (Keithley Instruments, Cleveland, OH, USA) was used to measure the current–voltage (I-V) characteristics. The external quantum efficiency (EQE) of the devices was calculated using the method described in the literature [51].

### 3.2. Materials

9*H*-carbazole (**1**), 2-methoxy-3-pyridinylboronic acid, 6-methoxy-3-pyridinylboronic acid, 2-ethylhexyl bromide, 1-bromoethane, 1-iodobutane, 2-amino-4-methylpyridine, tetrakis(triphenylphosphine)palladium(0), 18-crown-6, KOH, KI, KIO_3_, K_2_CO_3_, Cu, CuI, Na_2_SO_4_, acetone, acetic acid, isopropanol, chloroform, and dimethylformamide were purchased from Sigma-Aldrich and used as received.

3,6-diiodo-9*H*-carbazole (**2**) was prepared from commercially available 9*H*-carbazole (**1**) using S.H. Tucker’s iodination and crystallization procedure [52].

9-(2-ethylhexyl)-3,6-diiodocarbazole (**3**) was prepared through a standard 3,6-diiodo-9*H*-carbazole (**2**) alkylation reaction with 2-ethylhexyl bromide, as described earlier [53].

3-iodo-9*H*-carbazole (**4**) was prepared by the Tucker iodination method from 9*H*-carbazole [52].

3-(2-methoxypyridin-3-yl)-9*H*-carbazole (**5**) and 3-(6-methoxypyridin-3-yl)-9*H*-carbazole (**6**) were obtained in the Suzuki reaction using a previously described method [54].

9-butyl-3-iodocarbazole (**7**) and 9-ethyl-3-iodocarbazole (**8**) were obtained in a conventional alkylation process using 3-iodo-9*H*-carbazole (**4**) and 1-iodobutane or 1-bromoethane, respectively, as an alkylating agent [54].

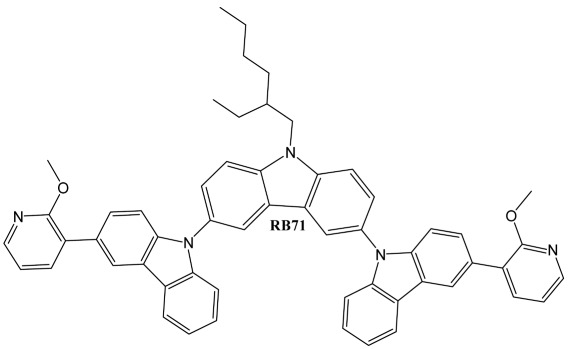



9-(2-ethylhexyl)-3,6-di [3-(2-methoxypyridin-3-yl)carbazol-9-yl]carbazole (**RB71**). 0.3 g (0.56 mmol) of 9-(2-ethylhexyl)-3,6-diiodocarbazole (**3**), 0.47 g (1.71 mmol) of 3-(2-methoxypyridin-3-yl)carbazole (**5**), and 0.014 g (0.05 mmol) of 18-crown-6 were refluxed under nitrogen in 10 mL of DMF. Then, potassium carbonate (0.31 g, 2.24 mmol), Cu (0.05 g, 0.78 mmol), and CuI (0.21 g, 1.10 mmol) were added stepwise to the solution. The mixture was refluxed for a further 24 h. After thin-layer chromatography (TLC) control, the inorganic materials were removed by filtration, and the organic fraction was extracted with chloroform. The extract was dried over anhydrous Na_2_SO_4_. The crude product was separated by silica gel column chromatography using an eluent, and the mixture of tetrahydrofuran and hexane (vol. ratio 1:5). Yield: 0.24 g (52%) of yellow powder. The ^1^H and ^13^C NMR spectra and MS spectra of compound are shown in Figures S1, S2 and S9. ^1^H NMR (400 MHz, CDCl_3_, δ, ppm): 8.25 (s, 2H, Ar), 8.18 (s, 2H, Ar), 8.12–8.09 (m, 4H, Ar), 7.67–7.65 (m, 2H, Ar), 7.60 (s, 4H, Ar), 7.54–7.52 (m, 2H, Ar), 7.35–7.32 (m, 4H, Ar), 7.24–7.18 (m, 4H, Ar), 6.95–6.92 (m, 2H, Ar), 4.31–4.25 (m, 2H, -CH_2_-), 3.93 (s, 6H, -CH_3_), 3.66 (s, 1H, -CH-), 1.50–1.41 (m, 8H, -CH_2_-) 0.98 (t, 3H, J = 7.6 Hz, -CH_3_), 0.87 (t, 3H, J = 7.2 Hz, -CH_3_). ^13^C NMR (101 MHz, CDCl_3_, δ, ppm): 161.1, 145.0, 142.3, 141.7, 141.3, 140.8, 138.8, 129.3, 127.3, 126.0, 123.2, 119.8, 117.8, 110.4, 109.9, 109.5, 61.8, 53.6, 39.7, 31.1, 28.9, 24.6, 23.1, 14.1, 11.0. MS (APCI+, 20 V): 824.77 ([M+H]^+^, 100%).

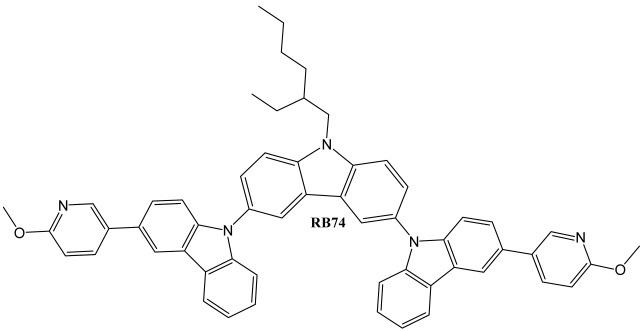



9-(2-ethylhexyl)-3,6-di [3-(6-methoxypyridin-3-yl)carbazol-9-yl]carbazole (RB74). 0.3 g (0.56 mmol) of 9-(2-ethylhexyl)-3,6-diiodocarbazole (**3**), 0.47 g (1.71 mmol) of 3-(6-methoxypyridin-3-yl)-9*H*-carbazole (**6**), and 0.014 g (0.05 mmol) of 18-crown-6 were stirred at reflux in DMF (10 mL) under nitrogen. Then, potassium carbonate (0.31 g, 2.24 mmol), Cu (0.05 g, 0.78 mmol), and CuI (0.21 g, 1.10 mmol) were added stepwise, and the resulting mixture was left to react for 24 h. After the reaction, the inorganic materials were removed by filtration, and the organic products were extracted using chloroform. The extract was dried over anhydrous Na_2_SO_4_. The objective product was separated in silica gel column chromatography using the mixture of tetrahydrofuran (THF) and hexane (vol. ratio 1:5) as an eluent. Yield: 0.24 g (52%) of light-yellow powder. The ^1^H and ^13^C NMR spectra and MS spectra of the compound are shown in Figures S3, S4 and S10. ^1^H NMR (400 MHz, δ, ppm): 8.41 (s, 2H, Ar), 8.20–8.11 (m, 6H, Ar), 7.84–7.82 (m, 2H, Ar), 7.59 (s, 4H, Ar), 7.48–7.46 (m, 2H, Ar), 7.38–7.31 (m, 6H, Ar), 7.26–7.21 (m, 2H, Ar), 4.34–4.24 (m, 2H, -CH_2_-), 3.92 (s, 6H, -CH_3_), 1.49–1.26 (m, 8H, -CH_2_-), 1.18 (s, 1H, -CH-), 0.98 (t, 3H, J = 7.2 Hz, -CH_3_), 0.86 (t, 3H, J = 7.2 Hz, -CH_3_). ^13^C NMR (101 MHz, CDCl_3_, δ, ppm): 163.1, 145.0, 142.3, 141.3, 140.8, 137.8, 129.2, 126.3, 125.0, 123.1, 119.9, 119.7, 110.7, 110.5, 110.3, 110.0, 53.6, 48.1, 40.0, 31.1, 28.9, 24.6, 23.2, 14.1, 11.1. MS (APCI+, 20 V): 824.74 ([M+H]^+^, 100%).

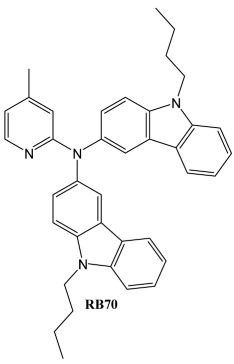



9-butyl-3-[*N*-(9-butylcarbazol-3-yl)-*N*-(4-methylpyridin-2-yl)amino]carbazole (RB70). 2.10 g (6.01 mmol) of 9-butyl-3-iodocarbazole (**7**), 0.5 g (4.62 mmol) of 2-amino-4-methylpyridine, and 0.09 g (0.10 mmol) of 18-crown-6 were stirred in 10 mL DMF at reflux under nitrogen. Then, potassium carbonate (1.93 g, 3.49 mmol), Cu (0.33 g, 3.46 mmol), and CuI (1.33 g, 3.49 mmol) were added stepwise. The mixture was left to react for 24 h. After TLC control, the inorganic materials were filtered out, and the organic product was extracted using chloroform. The extract was dried over anhydrous Na_2_SO_4_, and the crude product was purified by silica gel column chromatography using the mixture of tetrahydrofuran and hexane (vol. ratio 1:7) as an eluent. Yield: 0.39 g (18%) of white powder. The ^1^H and ^13^C NMR spectra and MS spectra of the compound are shown in Figures S5, S6 and S11. ^1^H NMR (400 MHz, δ, ppm): 8.03–8.02 (m, 1H, Ar), 7.95–7.89 (m, 4H, Ar), 7.38–7.29 (m, 7H, Ar), 7.18 (s, 1H, Ar), 7.08 (t, 2H, J = 7.6 Hz, Ar), 6.49–6.44 (m, 2H, Ar), 4.21 (t, 4H, J = 7.2 Hz, -CH_2_-), 2.09 (s, 3H, -CH_3_), 1.82–1.75 (m, 4H, -CH_2_-), 1.40–1.31 (m, 4H, -CH_2_-), 0.88 (t, 6H, J = 7.6 Hz, -CH_3_). ^13^C NMR (101 MHz, δ, ppm):141.0, 138.3, 125.8, 125.7, 123.7, 122.7, 120.6, 119.3, 118.7, 115.7, 111.9, 109.6, 108.8, 43.0, 31.2, 21.4, 20.6, 13.9. MS (APCI+, 20 V): 551.44 ([M+H]^+^, 100%).

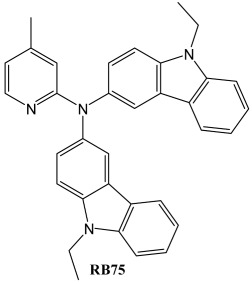



9-ethyl-3-[*N*-(9-ethylcarbazol-3-yl)-*N*-(4-methylpyridin-2-yl)amino]carbazole (**RB75**). 2.22 g (6.91 mmol) of 9-ethyl-3-iodocarbazole (**8**), 0.5 g (4.62 mmol) of 2-amino-4-methylpyridine, and 0.09 g (0.10 mmol) of 18-crown-6 were refluxed in DMF under nitrogen. Then, potassium carbonate (1.93 g, 3.49 mmol), Cu (0.33 g, 3.46 mmol), and CuI (1.33 g, 3.49 mmol) were added stepwise. The mixture was refluxed for 24 h. After the reaction and TLC control, the inorganic materials were filtered out, and the organic product was extracted using chloroform. The combined extract was dried over anhydrous Na_2_SO_4_. The crude product was purified by silica gel column chromatography using the mixture of THF and hexane (vol. ratio 1:10) as an eluent. Yield: 0.42 g (19%) of light-brown powder. The ^1^H and ^13^C NMR spectra and MS spectra of the compound are shown in Figures S7, S8 and S12. ^1^H NMR (400 MHz, δ, ppm): 8.06–7.90 (m, 5H, Ar), 7.39–7.30 (m, 7H, Ar), 7.19 (s, 1H, Ar), 7.10 (t, 2H, J = 7.2 Hz, Ar), 6.51–6.46 (m, 2H, Ar), 4.29 (q, 4H, J = 7.2 Hz, -CH_2_-), 2.11 (s, 3H, -CH_3_), 1.37 (t, 6H, J = 7.2 Hz, -CH_3_). ^13^C NMR (101 MHz, δ, ppm):140.5, 125.9, 125.5, 123.9, 122.7, 120.7, 119.4, 118.9, 109.6, 108.6, 37.7, 21.5, 13.9. MS (APCI+, 20 V): 495.28 ([M+H]^+^, 100%).

## 4. Conclusions

In summary, we report the synthesis and complete characterization of two series of potential host materials for phosphorescent organic light-emitting diodes, i.e., 9-(2-ethylhexyl)-3,6-di [3-(methoxypyridin-3-yl)carbazol-9-yl]carbazoles (**RB71** and **RB74**) and 9-alkyl-3-[*N*-(9-alkylcarbazol-3-yl)-*N*-(4-methylpyridin-2-yl)amino]carbazoles (**RB70** and **RB75**). The thermogravimetric analysis demonstrated that the new derivatives are highly thermally stable, with 5% weight loss temperatures ranging from 349 °C to 488 °C. Results from differential scanning calorimetry indicate that the new compounds may form amorphous layers, with glass transition temperatures varying between 68 °C and 173 °C, depending on the molecular weight of the new materials. The electroluminescent (EL) properties of the devices using some of the new hosts with 5.0, 10.0, 15.0, and 20.0 wt.% concentrations of dopant tris(2-phenylpyridine)iridium(III) were demonstrated. All the PhOLEDs emitted light from the green dopant with a maximum at about 515 nm and CIE coordinates of (0.30, 0.61). The formed devices with host **RB70** exhibited relatively low turn-on voltages of 3.5–5.4 V, maximum external quantum efficiencies of 4.8–8.1%, notably high maximal current efficiencies of 10.4–17.3 cd/A, and maximum brightness values exceeding 7400–11,200 cd/m^2^. The 15 wt.% Ir(ppy)_3_ emitter-based PhOLED with the host outperformed the other devices by displaying a maximum power efficacy of 9.6 lm/W, maximum current efficiency of 16.0 cd/A, and maximal external quantum efficiency of 6.7%, with a relatively low turn-on voltage of 3.7 eV as well as a luminance reaching 11,200 cd/m^2^. The efficiencies of the device appeared to be higher than those of previously reported OLEDs using the well-known 4,4′-bis(9-carbazolyl)-2,2′-biphenyl (CBP) host. It should be mentioned that the performances of the PhOLEDs could be further improved by thoroughly optimizing the layered structures, formation conditions, and charge-transporting materials.

## Figures and Tables

**Table 1:** Characteristics of OLEDs using host material **RB71** with different concentrations of emitter Ir(ppy)_3_.

**Host**	**Dopant Ir(ppy)_3_, wt. %**	**Turn-on voltage, V**	**Power efficacy (PE), lm/W**	**Current efficacy (CE), cd/A**	**EQE, %**	**CIE (x, y)**	**Maximum luminance L_MAX_, cd/m^2^**
			*@100, 1000 cd/m^2^ and maximum*			*@100, 1000 cd/m^2^*	
**RB71**	5	7.2	5.8/4.4/5.8	15.1/13.5/15.6	4.3/3.6/6.1	(0.29, 0.61)/(0.29, 0.61)	3307
	10	5.6	7.2/5.3/7.3	15.1/13.2/15.3	4.6/3.8/5.8	(0.30, 0.62)/(0.30, 0.61)	3628
	15	5.0	1.2/4.2/4.2	3.1/11.4/11.4	1.9/4.8/4.8	(0.30, 0.62)/(0.30, 0.62)	5795
	20	4.3	7.9/5.2/8.0	12.7/10.5/12.7	3.8/2.3/5.4	(0.30, 0.62)/(0.31, 0.62)	4090

**Table 2:** Characteristics of OLEDs using host material **RB70** with different concentrations of emitter Ir(ppy)_3_.

**Host**	**Dopant Ir(ppy)_3_, wt. %**	**Turn-on voltage, V**	**Power efficacy (PE), lm/W**	**Current efficacy (CE), cd/A**	**EQE, %**	**CIE (x, y)**	**Maximum luminance L_MAX_, cd/m^2^**
			*@100, 1000 cd/m^2^ and maximum*			*@100, 1000 cd/m^2^*	
**RB70**	5	5.4	8.0/5.6/8.0	16.1/14.0/16.5	5.3/4.3/5.3	(0.29, 0.60)/(0.29, 0.60)	7420
	10	4.6	4.6/7.9/9.1	8.1/16.5/17.4	2.5/4.8/4.8	(0.30, 0.61)/(0.30, 0.61)	9944
	15	3.7	8.7/8.9/9.6	12.5/15.9/16.0	4.0/4.7/6.7	(0.30, 0.62)/(0.30, 0.61)	11,200
	20	3.5	6.6/5.8/6.6	8.9/10.2/10.4	2.8/3.0/8.1	(0.30, 0.62)/(0.30, 0.62)	10,800

## Data Availability

All data produced or examined in this study are provided within this published article.

## Supplementary Materials

The following supporting information can be downloaded at: https://www.mdpi.com/article/10.3390/molecules30091911/s1, Figure S1: ^1^H NMR spectrum of RB71 in CDCl_3_; Figure S2: ^13^C NMR spectrum of RB71 in CDCl_3_; Figure S3: ^1^H NMR spectrum of RB74 in CDCl_3_; Figure S4: ^13^C NMR spectrum of RB74 in CDCl_3_; Figure S5: ^1^H NMR spectrum of RB70 in CDCl_3_; Figure S6: ^13^C NMR spectrum of RB70 in CDCl_3_; Figure S7: ^1^H NMR spectrum of RB75 in CDCl_3_; Figure S8: ^13^C NMR spectrum of RB75 in CDCl_3_; Figure S9: MS spectra of RB71 compound; Figure S10: MS spectra of RB74 compound; Figure S11: MS spectra of RB70 compound; Figure S12: MS spectra of RB75 compound; Figure S13: Fluorescence spectra of RB compounds in THF solutions; Figure S14: Fluorescence spectra of RB compounds from thin films; Figure S15: CV curve of RB70 compound; Figure S16: CV curve of RB71 compound.
