# Supplementary material for: Carbazolyl Electron Donor and Pyridinyl Electron Acceptor Containing Derivatives as Potential Host Materials for Green Organic Light-Emitting Diodes
Source: Molecules. 2025 Apr 25;30(9):1911. doi: 10.3390/molecules30091911 (PMC12073105; doi:10.3390/molecules30091911)
Supplement: Supplementary file 1 [file molecules-30-01911-s001.zip › molecules-3584893-supplementary.pdf]

Supporting information

Carbazolyl Electron Donor and Pyridinyl Electron Acceptor Containing Derivatives as Potential Host Materials for Green Organic Light Emitting Diodes

Raminta Beresneviciute[1, †], Anil Kumar[2, †], Dovydas Blazevicius[1], Sushanta Lenka[2], Song-Ting Hsieh[3], Ming-Feng Tsai[2], Gintare Krucaite[1], Daiva Tavgeniene[1], Jwo-Huei Jou[2*] and Saulius Grigalevicius[1*]

[1]Department of Polymer Chemistry and Technology, Kaunas University of Technology, Radvilenu Plentas 19, LT50254 Kaunas, Lithuania
[2]Department of Materials Science and Engineering, National Tsing Hua University, No. 101, Section 2, Guangfu Rd., East District, Hsinchu, Taiwan, 30013
[3]Department of Chemistry, National Tsing Hua University, No. 101, Section 2, Guangfu Rd., Hsinchu 30013, Taiwan
[†] These authors contributed equally to this work.
* Correspondence: authors e-mails: saulius.grigalevicius@ktu.lt and jjou@mx.nthu.edu.tw

List of Figures

Figure S1. ^{1}H NMR spectrum of RB71 in $CDCl_3$
Figure S2. ^{13}C NMR spectrum of RB71 in $CDCl_3$
Figure S3. ^{1}H NMR spectrum of RB74 in $CDCl_3$
Figure S4. ^{13}C NMR spectrum of RB74 in $CDCl_3$
Figure S5. ^{1}H NMR spectrum of RB70 in $CDCl_3$
Figure S6. ^{13}C NMR spectrum of RB70 in $CDCl_3$
Figure S7. ^{1}H NMR spectrum of RB75 in $CDCl_3$
Figure S8. ^{13}C NMR spectrum of RB75 in $CDCl_3$
Figure S9. MS spectra of RB71 compound
Figure S10. MS spectra of RB74 compound
Figure S11.MS spectra of RB70 compound
Figure S12. MS spectra of RB75 compound
Figure S13. Fluorescence spectra of RB compounds in THF solutions
Figure S14. Fluorescence spectra of RB compounds from thin films
Figure S15. CV curve of RB70 compound
Figure S16. CV curve of RB71 compound

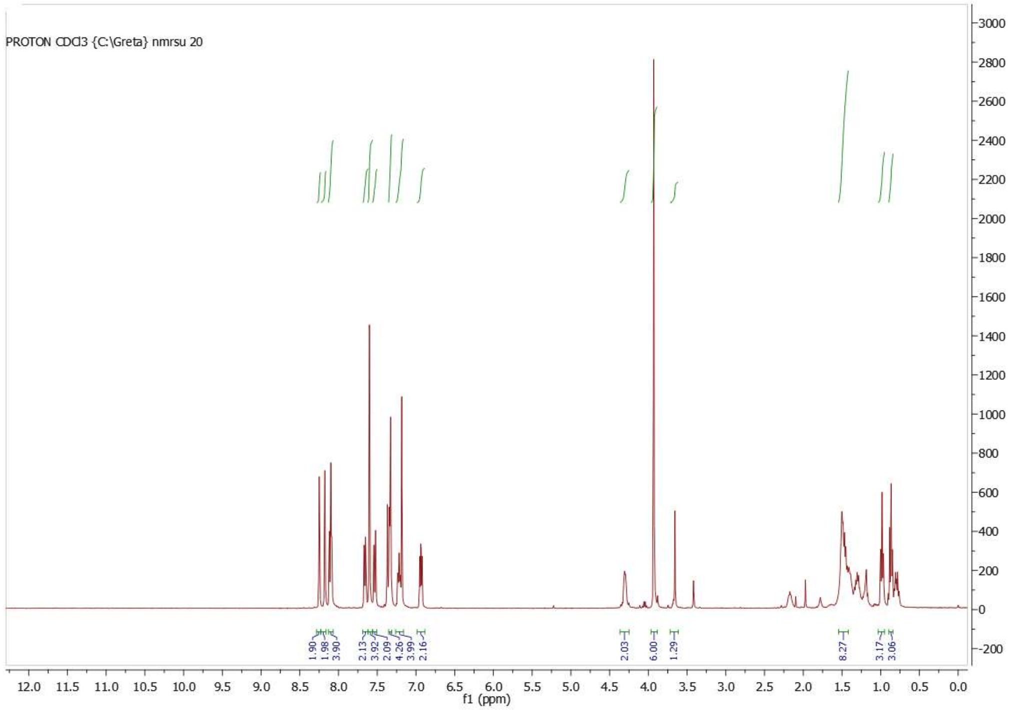

Figure S1. ^{1}H NMR spectrum of RB71 in $CDCl_3$

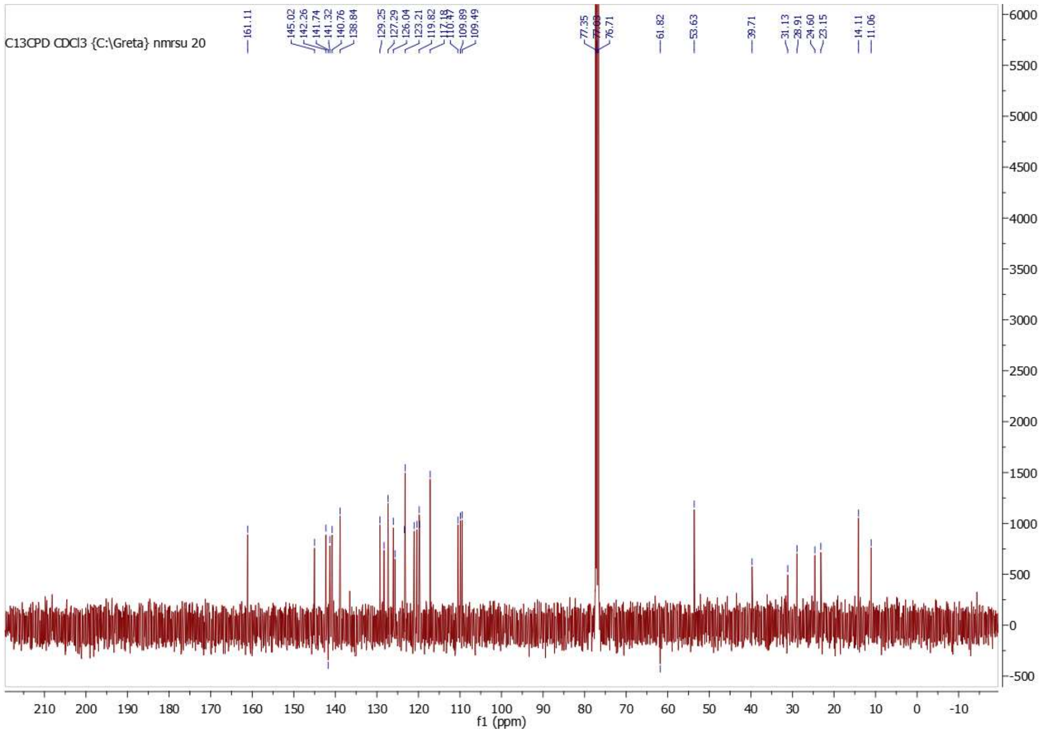

Figure S2. ^{13}C NMR spectrum of RB71 in $CDCl_3$

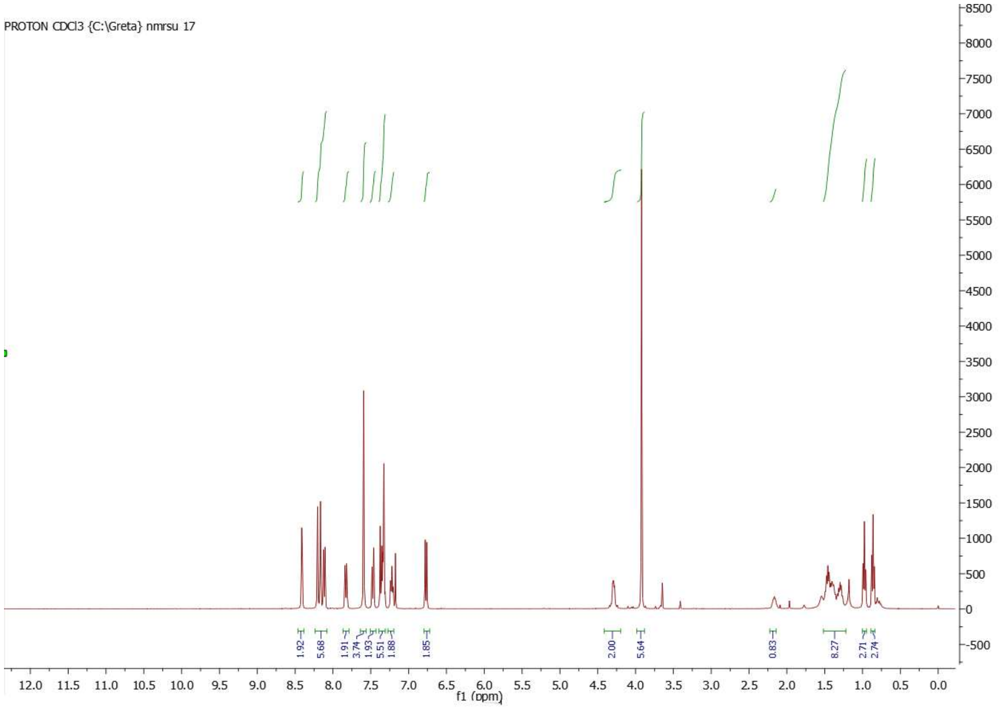

Figure S3. ^{1}H NMR spectrum of RB74 in $CDCl_3$

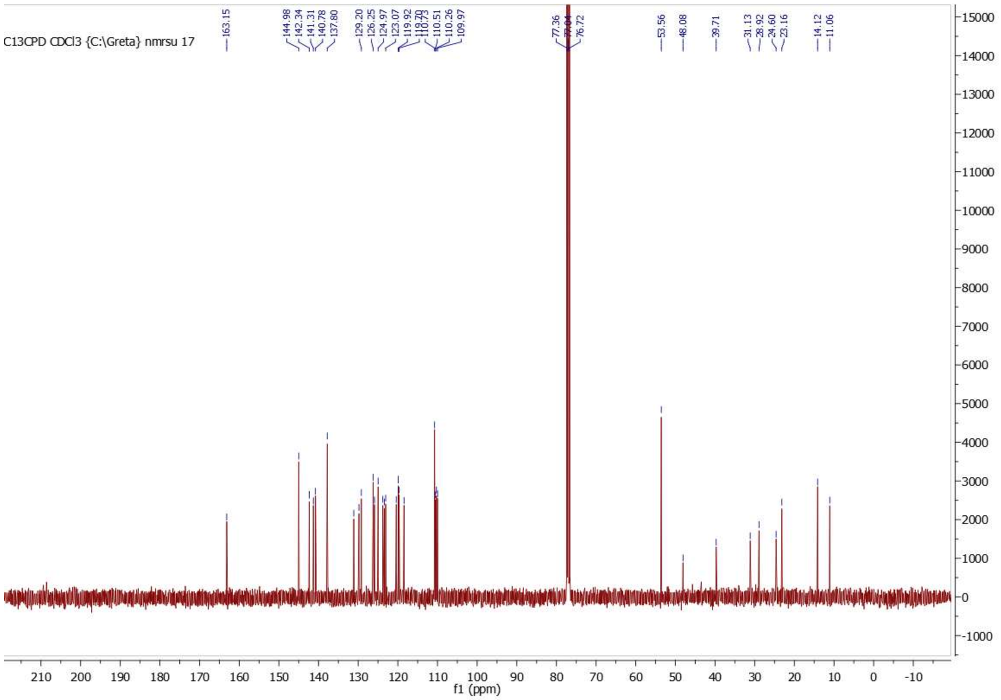

Figure S4. ^{13}C NMR spectrum of RB74 in $CDCl_3$

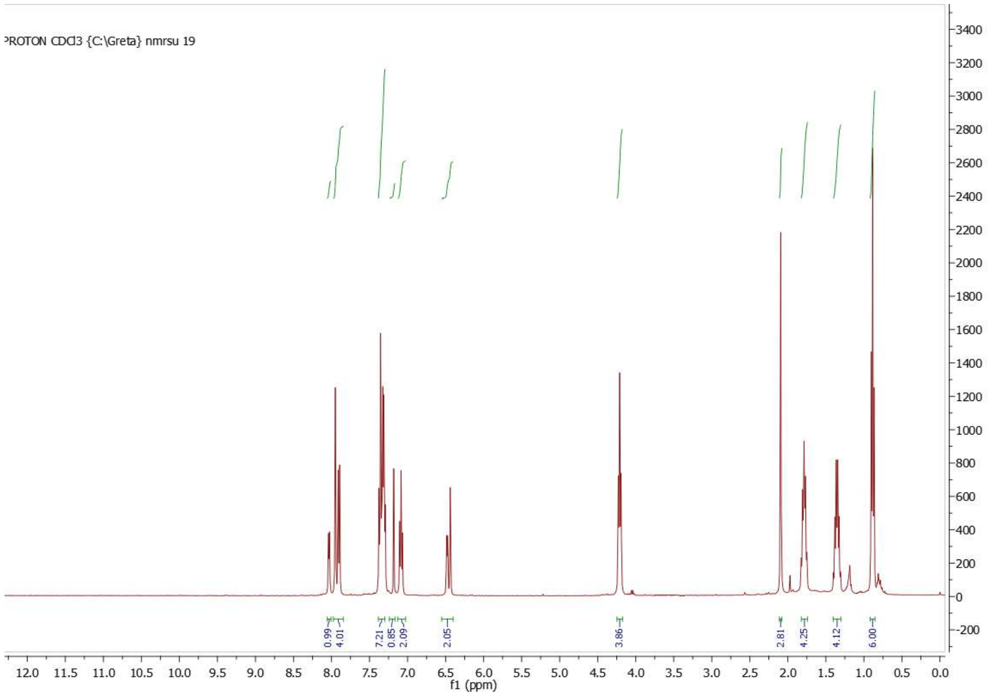

Figure S5. ^{1}H NMR spectrum of RB70 in $CDCl_3$

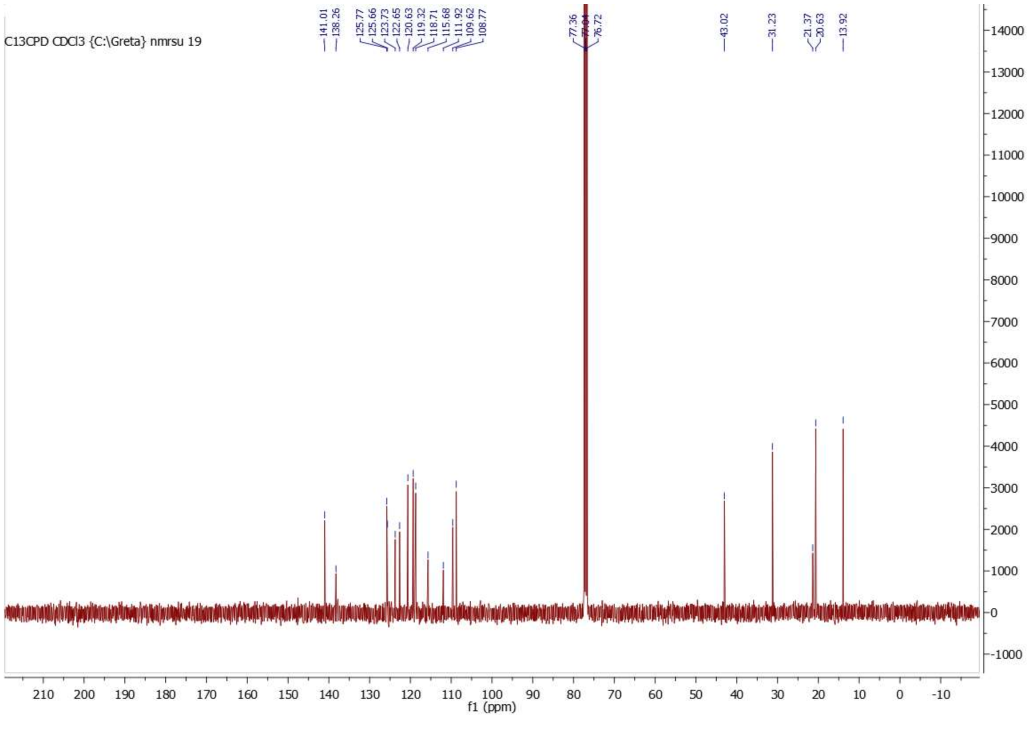

Figure S6. ^{13}C NMR spectrum of RB70 in $CDCl_3$

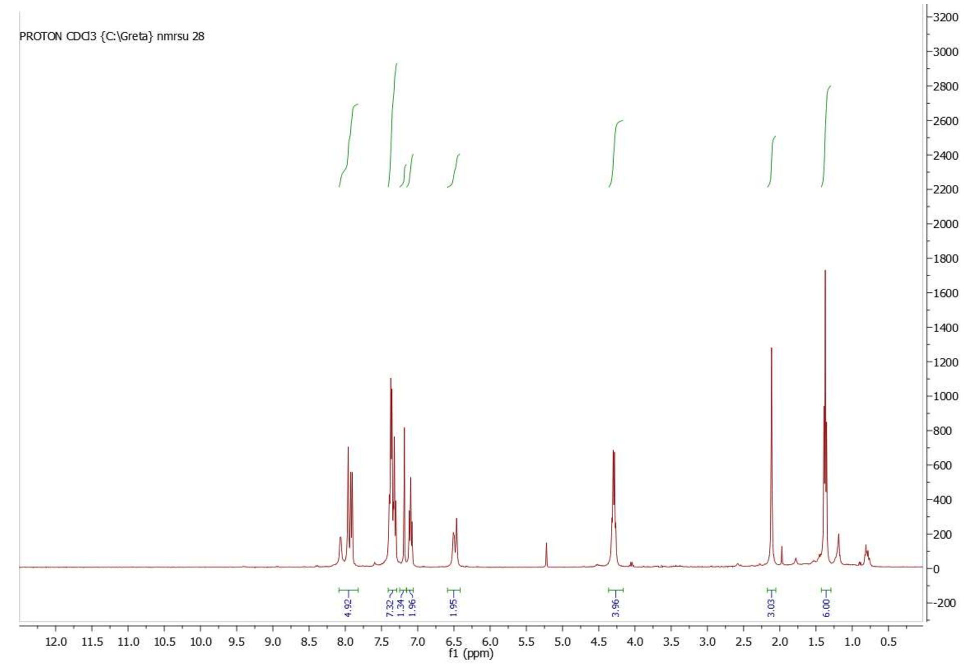

Figure S7. ^{1}H NMR spectrum of RB75 in $CDCl_3$

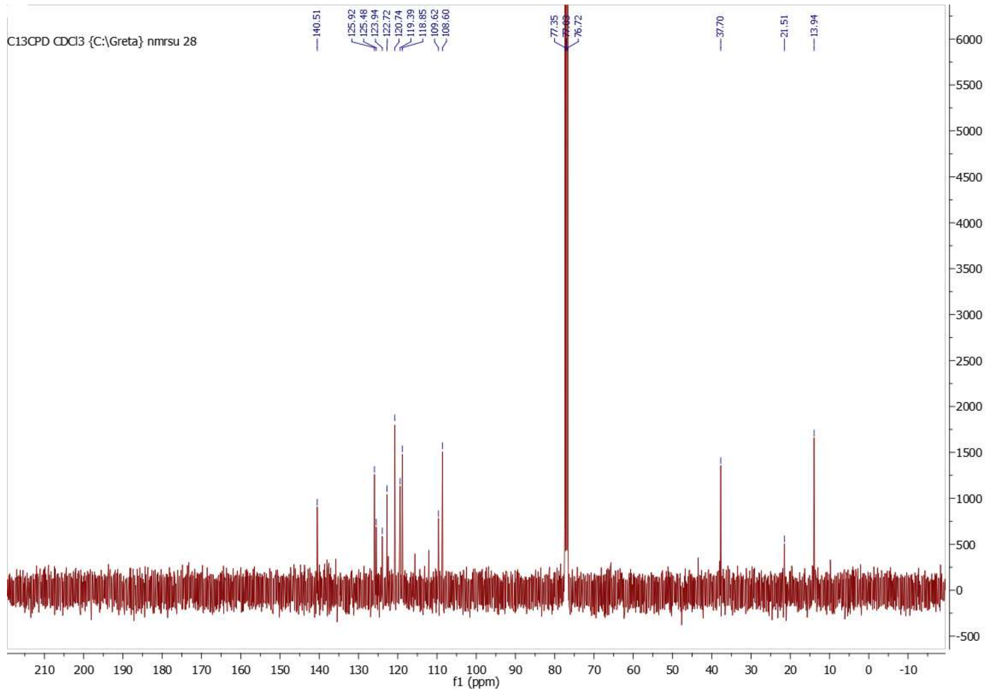

Figure S8. ^{13}C NMR spectrum of RB75 in $CDCl_3$

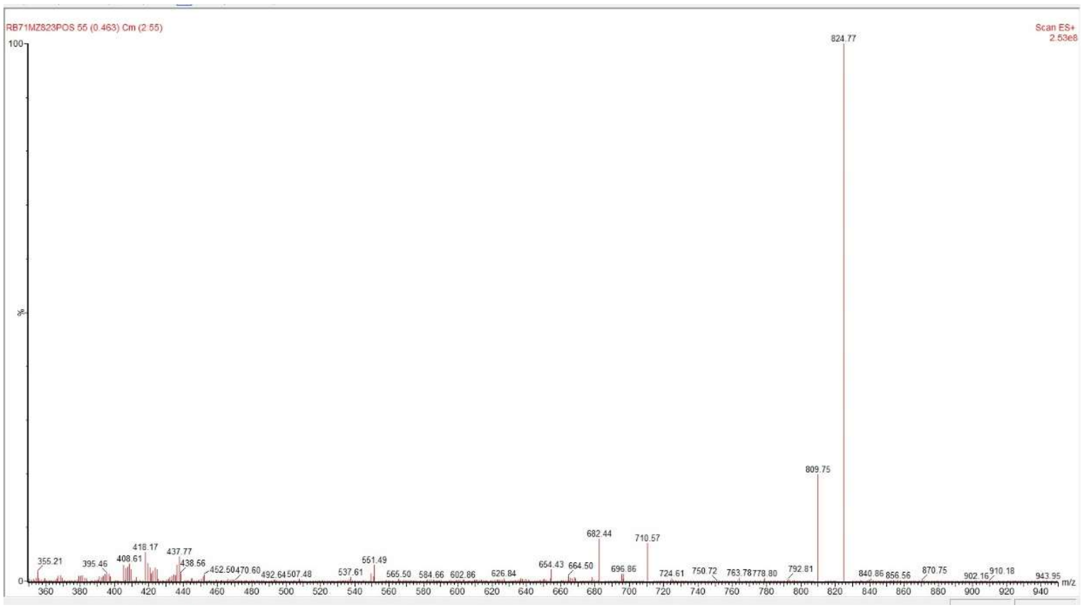

Figure S9. MS spectra of RB71 compound

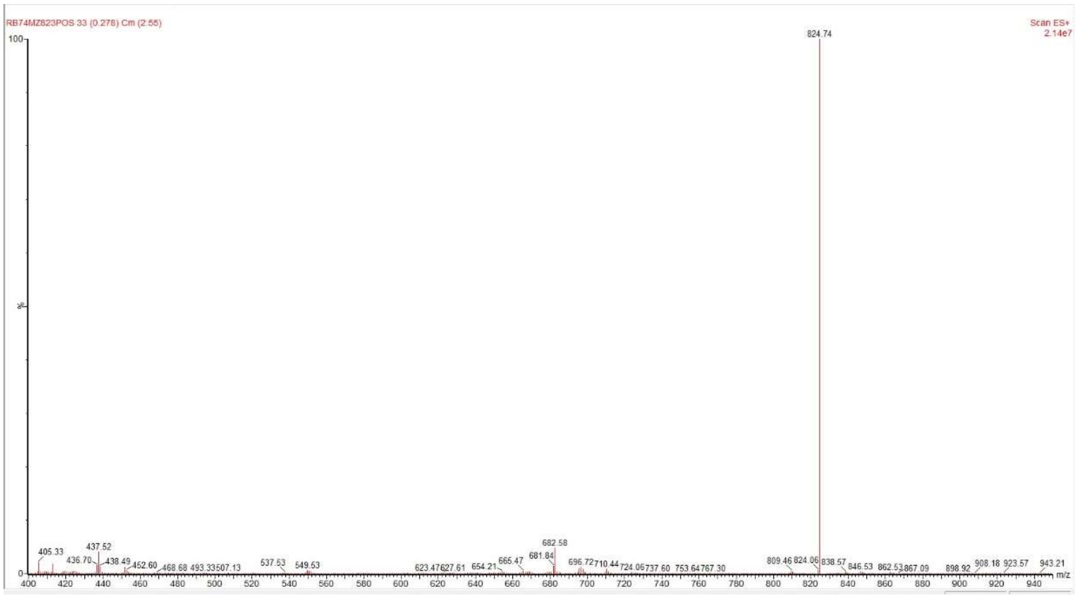

Figure S10. MS spectra of RB74 compound

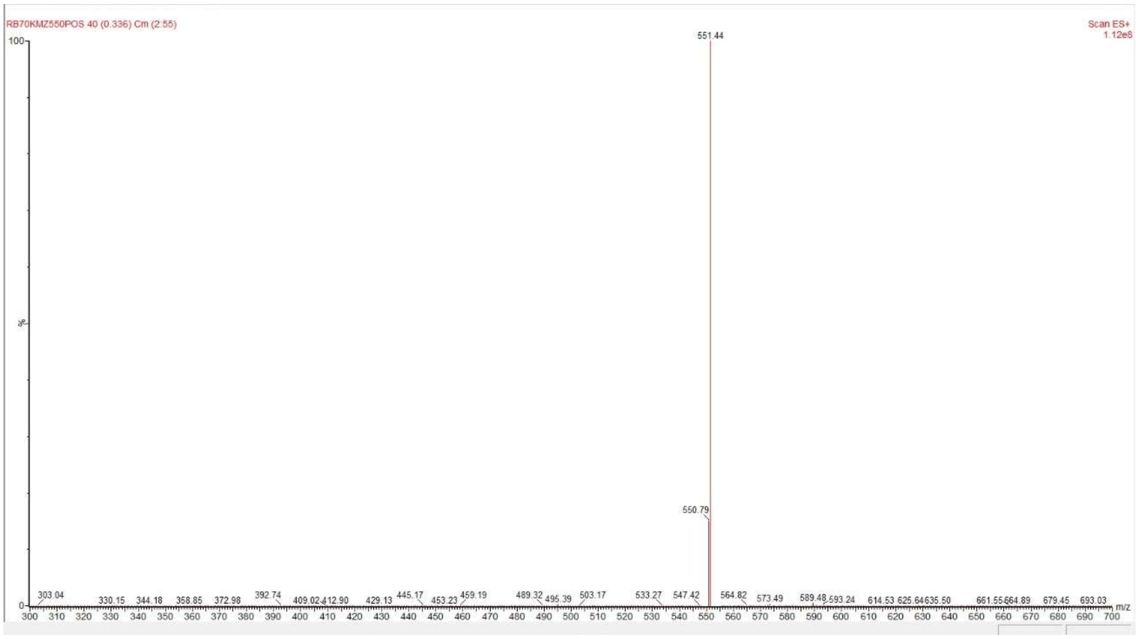

Figure S11.MS spectra of RB70 compound

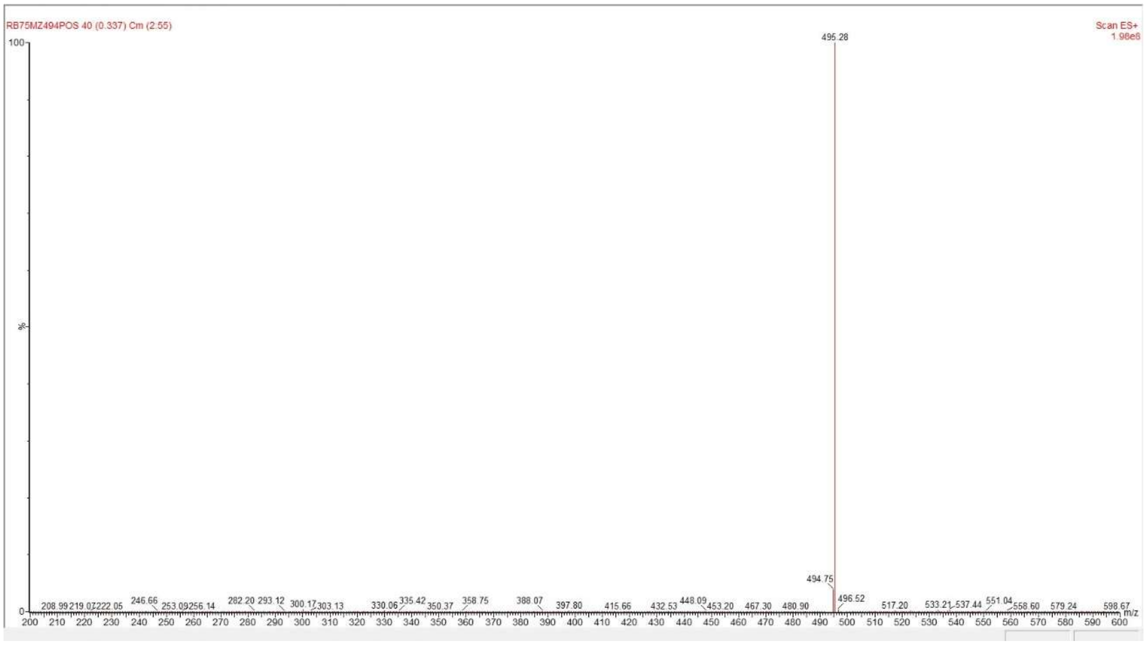

Figure S12. MS spectra of RB75 compound

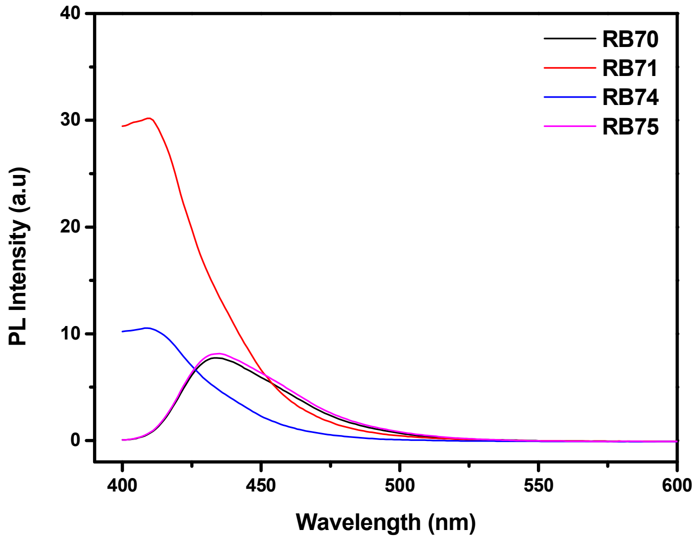

Figure S13. Fluorescence spectra of RB compounds of their THF solutions

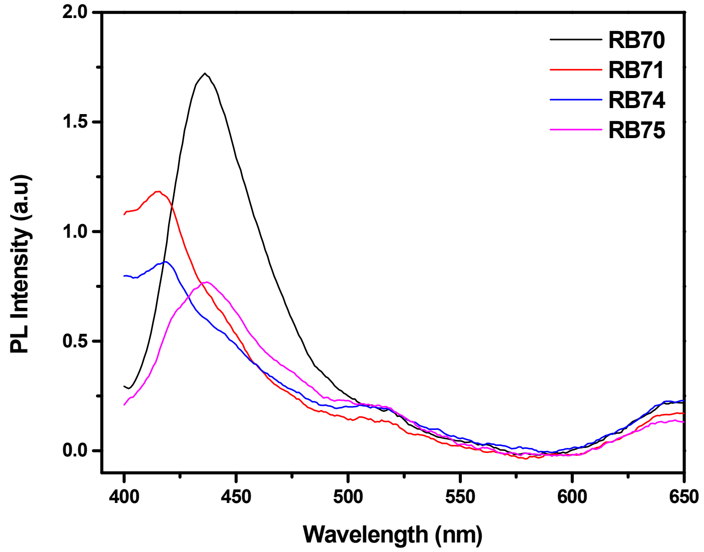

Figure S14. Fluorescence spectra of RB compounds from thin films

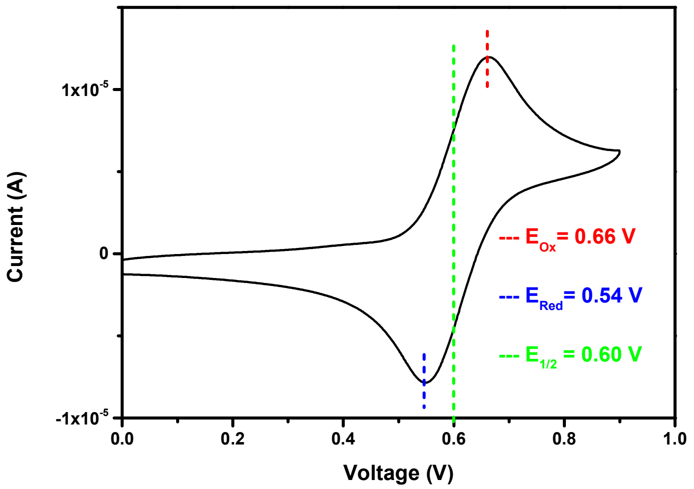

Figure S15. CV curve of RB70 compound

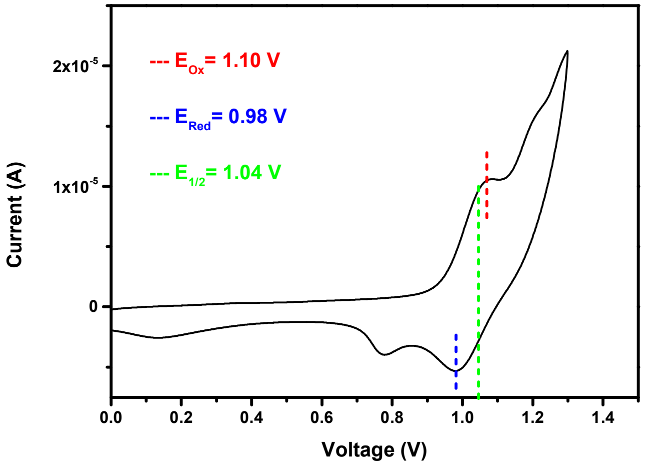

Figure S16. CV curve of RB71 compound
